# Can heparin therapy reduce 28-day mortality in adult severe sepsis patients?

**DOI:** 10.1186/s13054-015-0768-1

**Published:** 2015-02-19

**Authors:** Li-bing Jiang, Yue-feng Ma, Xia Feng, Mao Zhang

**Affiliations:** Department of Emergency Medicine, Second Affiliated Hospital, School of Medicine & Institute of Emergency Medicine, Zhejiang University, Jiefang Road 88, Hangzhou, 310009 China; The Second Department of Respiration, Affiliated Hospital of Zunyi Medical College, Dalian Road 149, Zunyi, Guizhou 563000 China

A study by Wang and colleagues [1] in a previous issue of *Critical Care* shows that heparin may reduce 28-day mortality in severe sepsis patients without causing an increased risk of bleeding. Therefore, the authors recommend the use of heparin for sepsis and severe sepsis. Although their result may significantly influence clinical practice, several confounding factors should be noticed.

In this study, eight studies performed analyses of mortality and seven studies performed analyses of bleeding events. From Additional file two, three non-randomized controlled trials (NRCTs) account for 88.42% of weight on mortality [2-4]; and from Figure three, two NRCTs account for 92.27% of weight on bleeding events [2,3]. In other words, the results of this meta-analysis are almost equivalent to those of the meta-analysis of these five NRCTs. Given the above analysis, the subgroup analyses in this study were unnecessary. However, the three studies were only placebo groups in three large multi-center randomized controlled trials [2-4]. Patients in these placebo groups were not randomly assigned to receive heparin or other therapy. In addition, it is possible that patients receiving heparin were less severe, because the Acute Physiology and Chronic Health Evaluation II score was not reported in these placebo groups. Moreover, as the initial times of heparin treatment were not defined clearly in the above three studies, we cannot rule out the possibility that patients who had died early would have no chance to receive heparin, whereas patients who had a longer survival would be more inclined to receive heparin. Finally, the dose of heparin in the three trials, and within each trial, was not controlled.

## Abbreviation

NRCT: Non-randomized controlled trial
